# Synthesis, Crystal Structure, and Magnetic Properties of a New Mixed Metal (Co(II), Ni(II)) Cubane

**DOI:** 10.3390/ma10020178

**Published:** 2017-02-14

**Authors:** Ramadan Mohamed Elmehdawi, Mohamed Nasir EL-Kaheli, Ramadan Gamodi Abuhmaiera, Fathia Ali Treish, Mufida El Mabruk Ben Younes, Carla Bazzicalupi, Annalisa Guerri, Andrea Caneschi, Asma Amjad

**Affiliations:** 1 Chemistry Department, Tripoli-University, Tripoli, Libya; manskahl@yahoo.com (M.N.E.-K.); Rouhmaa@yahoo.com (R.G.A.); Treish@gmail.com (F.A.T.); mbenyouns@yahoo.com (M.E.M.B.Y.); 2 Chemistry Department, “U. Schiff”, University of Florence, Sesto Fiorentino (Fl) 50019, Italy; annalisa.guerri@unifi.it (A.G.); a.amjad@knights.ucf.edu (A.A.); 3 INSTM-Research Unit at University of Florence, Sesto Fiorentino (Fl) 50019, Italy

**Keywords:** mixed Co/Ni, cubane, Schiff base, crystal structure, magnetic properties

## Abstract

The mixed Co(II)/Ni(II) complex, [Co_2.67_Ni_1.33_L_4_(CH_3_COO)_2_][BPh_4_]_2_·0.75H_2_O where HL = 4-(salicylaldimine)antipyrine, was isolated as an orange solid from the reaction of 4-(salicylaldimine)antipyrine, with mixed cobalt(II) acetate and nickel(II) acetate in ethanol. The complex was characterized by Frustrated Total Internal Reflection (FTIR), UltraViolet Visible spectroscopy (UV-Vis), X-ray single crystal diffraction, and by elemental analysis. The complex is composed of two symmetry independent cationic units, A and B. The two units are essentially isostructural; nevertheless, small differences exist between them. The units contain four metal atoms, arranged at the corners of a distorted cubane-like core alternately with phenoxy oxygen of the Schiff base. The overall eight corners occupied by metal ions in the asymmetric unit are shared between cobalt and nickel in a 5.33:2.67 ratio. Each metal divalent cation binds three coordinated sites from the corresponding tridentate Schiff base ligand, the fourth one is bound by the acetate oxygen, the fifth and the sixth donor sites come from the phenolate oxygens of other Schiff base ligands. Intermolecular hydrogen bonds join the complexes to the water molecules present in the crystal packing. The magnetic characterization was carried out for this new complex and for its isostructural counterpart containing only cobalt ions. The magnetic measurements for the cobalt(II)/nickel(II) mixed compound indicate either antiferromagnetic interactions among the two cubanes or an anisotropic contribution, whereas a ferromagnetic interaction is observed within the cubane, for both the complexes, as expected by geometrical considerations. A comparison between the magnetic properties of the pure cobalt(II) derivative and similar systems discussed in literature, is presented.

## 1. Introduction

**Scheme 1:**
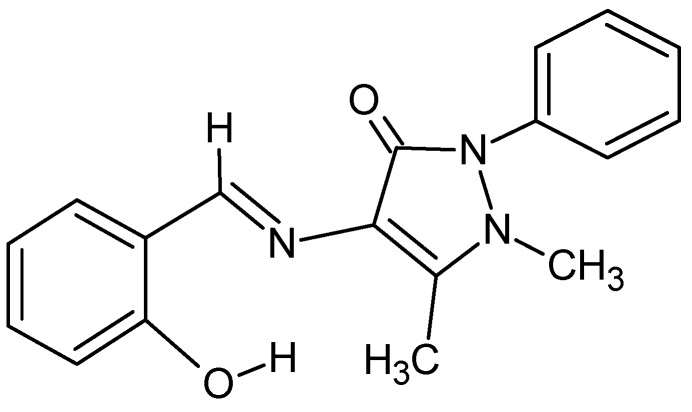
Schiff base ligand (HL).

Poly transition metal compounds with cube-shape, generally called cubane, especially for first-row transition elements have attracted considerable attention. In recent years, the magnetic properties of cubane complexes of first row transition elements have been studied, mainly because seminal single-molecule magnet (SMM), [Mn_12_O_12_(OAc)_16_(H_2_O)_4_], has a cubane ${\mathrm{Mn}}_{4}O_{4}$ center [1,2,3]. The first cobalt-based SMM was a cubane with deprotonated hydroxymethylpyridine ligands [4], followed by Murrie et al. who described a cobalt-based SMM with citrate ligands [5]; Falvello and coworkers also reported the structure of a square anionic two-dimensional citrate cubane [Cs[Co(H_2_O)_6_]{[Co_4_(C_6_H_4_O_7_)][μ-Co(H_2_O)_4_]_2_·12H_2_O} [6]; in the follow cobalt(II) ion was also the main actor of the single-chain magnets (SCM) class of compounds [7,8]. Moreover, transition metal complexes with cubane-like ${\mathrm{Ni}}_{4}O_{4}$ tetranuclear complexes have been extensively studied since the first report by Andrew and Blake in 1969 of a ferromagnetic interaction that lead to a S = 4 ground state [9,10]. The discovery of single-molecule magnetism in nickel(II) and cobalt(II) high spin molecular tetranuclear compounds and particularly in cubane-like ${\mathrm{Ni}}_{4}$ tetranuclear complex revived the correlation between the magnetic anisotropy of the high spin ground state and the relaxation of the magnetization at low temperature [11]. The exchange interaction in the tetranuclear cubane-like complexes is well understood, especially in nickel(II) complexes. Ferromagnetic interaction correlates to the Ni–O–Ni angle within the cubane, being active only when the Ni-O-Ni angle is lower than 98°. Such angular values were found for ${\mathrm{Co}}_{4}$-tetranuclear cubane cores complex with the general formula [${\mathrm{Co}}_{4}L_{4}{\left({\mathrm{CH}}_{3}\mathrm{COO}\right)}_{2}{]}_{2}{\left[{\mathrm{BPh}}_{4}\right]}_{4}\cdot 0.5H_{2}O$ where HL is 4-(salicylaldimine)antipyrine (Scheme 1) [12]. This type of Schiff base which can act as bi and tri-dentate ligand often forms polynuclear complexes with cubane core structure where the phenoxy oxygen acts as a good bridging group. Thus, such ligands can be used in the tactical synthesis of metal-assembled complexes with a cubane-like core as well as other salicylidenemine and salicylideneimine ligands (Scheme 1), which are known to give complexes containing either ${\mathrm{Co}}_{4}O_{4}$ or ${\mathrm{Ni}}_{4}O_{4}$ a cubane-like core. Here, we report the synthesis, structure, and magnetic properties of a cobalt(II)/nickel(II) mixed cubane-like polynuclear complex containing the 4-(salicylaliminato)antipyrine Schiff base acting as a tridentate ligand. Magnetic properties of the counterpart pure cobalt(II)-complex are reported for comparison.

## 2. Material and Measurement

All reagent grade chemicals used in this work were obtained commercially from Aldrich or BDH and used without any further purification. All manipulations were carried out under atmospheric pressure. Elemental analysis was performed on a Vario El(III) elemental analyzer (C, H, and N) (Bruker Analytik Gmbhg, Hanau, Germany) and on an Inductively Coupled Plasma Atomic Emission Spectroscopy (ICP-AES) Varian ES720 (Varian, Sesto Fiorentino, Italy) and an Inductively Coupled Plasma Mass Spectroscopy (ICP-MS) Thermofisher Element II (Co and Ni) (Varian, Sesto Fiorentino, Italy). Frustrated Total Internal Reflection (FTIR) spectra were recorded at room temperature with a Bruker IFS-25 OPUS/IR (Bruker Analytik Gmbhg, Hanau, Germany) over 400 to 4000 ${\mathrm{cm}}^{-1}$ range with resolution of 4 cm^−1^. The electronic absorption spectrum was recorded over the range 200 to 920 nm using Perkin Elmer Lambda 25, UltraViolet Visible (UV-Vis) spectrophotometer (PerkinElmer Life and Analtical Science, Shelton, CT, USA). Dc temperature dependent magnetic measurements were conducted on a Quantum Design MPMS SQUID magnetometer (Quantum Design, Sesto Fiorentino, Italy), Ac magnetic susceptibility measurements were carried out on a Quantum Design PPMS (Quantum Design, Sesto Fiorentino, Italy) in ac mode at both zero and applied external dc field up to 1 KOe in the presence of 5 Oe oscillating magnetic field over a wide range of frequencies from 10 Hz to 10 kHz. The diamagnetic contribution from the sample holder and samples estimated using the Pascal’s method (−2.513 × 10^−3^ emu·mol^−1^) were corrected. The compounds were gently ground to thin powder and pressed in pellets using a 4 mm diameter dye.

### 2.1. Synthesis of (4-Salicylaldimine)antipyrine-EtOH (HL·EtOH)

Yellow microcrystals of Schiff base, HL, were prepared using previously described method [13,14]. The Schiff base was characterized by FTIR, single crystal X-ray diffraction, and by elemental analysis. Analytically calculated for C_20_H_23_N_3_O_3_: C, 67.96; H, 6.57; N, 11.89; Found: C, 67.95; H, 6.25; N, 11.70.

#### 2.1.1. Synthesis of [${\mathrm{Co}}_{5.33}{\mathrm{Ni}}_{2.67}L_{8}{\left({\mathrm{CH}}_{3}\mathrm{COO}\right)}_{4}]{\left[{\mathrm{BPh}}_{4}\right]}_{4}\cdot 1.5H_{2}O\;\left(1\right)$

The title complex (**1**) was prepared using previously described method [12]. A solution of HL (2.0 mmol, 0.60 g) in 15 ml ethanol, cobalt(II) acetate (1.0 mmol) and Ni(II) acetate (1.0 mmol) in 15 mL ethanol were mixed and refluxed for about 2 h. After that time, an orange solid material precipitated from the solution. The resulting solid was separated by filtration, washed with ethanol and dichloromethane, and air dried. The product was dissolved in hot methanol and a suitable methanol solution of sodium tetraphenylborate (1.0 g in 10 mL methanol) was added and stirred for a while, to give the title complex as an orange solid. The final product was dissolved in acetone and the resulting solution filtered; on slow evaporation, red solid like crystals were formed (yield 62%). The final product was characterized by FTIR, UV-Vis, single crystal X-ray diffraction and by elemental analysis (C, H, N, analysis for the organic part and ICP for Ni and Co). Analytically calculated for C_248_H_223_B_4_Co_5.33_Ni_2.67_N_24_O_25.50_: C, 66.76; H, 5.05; N, 7.54; Co, 7.04; Ni, 3.51 Found: C, 67.62; H, 5.55; N, 6.90, Co, 6.55; Ni, 3.27.

#### 2.1.2. Synthesis of [${\mathrm{Co}}_{4}L_{4}{\left({\mathrm{CH}}_{3}\mathrm{COO}\right)}_{2}{]}_{2}{\left[{\mathrm{BPh}}_{4}\right]}_{4}.0.5H_{2}O$ (**2**)

Red solid of (**2**) was prepared using the previously described method [12]. A solution of HL (2.0 mmol, 0.60 g) in 15 mL ethanol and cobalt(II) acetate (2.0 mmol) in 15 mL ethanol were refluxed together; after 2 h, a red solid material was precipitated. The resulting solid was separated by filtration, washed with ethanol and dichloromethane, and air-dried. The product was dissolved in hot methanol and a suitable methanol solution of sodium tetraphenylborate was added and stirred for a while to obtain a red solid. The final product was dissolved in acetone and filtered; on slow evaporation, red crystals of (**2**) were formed (yield 62%). The final product was characterized by elemental analysis, FTIR, UV-Vis, and single-crystal X-ray diffraction analysis [12].

### 2.2. Crystal Structures Determination and Refinement 

Reflection data for the X-ray analyses were collected at 100 K on an Oxford Diffraction Xcalibur, Sapphire3 diffractometer (HL) (Oxford Diffraction, Oxford, UK) and on an Oxford Diffraction XcaliburPX diffractometer (Oxford Diffraction, Oxford, UK). Both diffractometers were equipped with CCD area detector and Mo Kα radiation (0.71073 Å). Data collections, reductions, and absorption corrections (SCALE3 ABSPACK routine) were carried out through the suite CrysAlysPro (Oxford Diffraction Ltd.: Oxfordshire, UK) [15]. Both structures were solved by direct methods of the SHELXD program [16] and then refined by full-matrix least squares against F2 using all data (SHELX-2014, University of Göttingen, Göttingen, Germany) [17]. Molecular plots were produced with Ortep-3 for windows program (Farrugia, 1997, University of Glasgow, Glasgow, Scotland) [18], and geometrical calculations were performed with the program PARST [19]. CIF files are available from the Cambridge Crystallographic Data Center (CCDC code1520222 (HL) and 991790 for (**1**)).

#### 2.2.1. Details of the Crystal Structure Determination and Refinement of HL

Suitable yellow single crystals of the ligand HL were obtained by slow cooling of a diluted hot ethanolic solution. A prism-shaped crystal of proper size was selected for data collection. All non-hydrogen atoms were anisotropically refined. Phenolic hydrogen was found in the ΔF map and freely refined with isotropic displacement parameter. All the other hydrogen atoms were introduced in calculated positions with isotropic displacement parameters refined accordingly to the linked atoms. The details of the crystallographic data and structure refinement of HL are shown in Table S1.

#### 2.2.2. Details of the Crystal Structure Determination and Refinement of (**1**)

Suitable red single crystals of the complex were obtained by slow diffusion of ether into an acetone solution at ambient temperature. A prism shaped crystal of the appropriate size was selected for data collection. The eight corners of the two cubane-like $M_{4}O_{4}$ cores were shared by cobalt and nickel ions. The occupancy factors for cobalt and nickel ions sharing the same vertex were freely refined and their sum was fixed to one. All non-hydrogen atoms were anisotropically refined. The OW1 water molecule was disordered and it was refined using a fractional population parameter site occupancy factors (s.o.f. = 0.5). Positions of hydrogen atoms bonded to the water molecules were not localized in the ΔF map.

The disorder affecting the tetranuclear compounds’ vertices, together with the very high number of atoms contained in the asymmetric unit and low number of observed reflection (15,114 reflections with *I* > 2*σ*(*I*)) can explain the values obtained at the end of refinement for goodness-of-fit and agreement factors. Several different crystallization trials were checked to choose the best performing sample. The details of the crystallographic data and structure refinement of (**1**) are shown in Table 1.

## 3. Results and Discussion

**Scheme 2:**
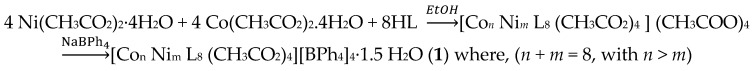
Synthesis of the title complex.

A new mixed cobalt(II) and nickel(II) complex ${\mathrm{Co}}_{5.33}{\mathrm{Ni}}_{2.67}L_{8}{\left({\mathrm{CH}}_{3}\mathrm{COO}\right)}_{4}]{\left[{\mathrm{BPh}}_{4}\right]}_{4}.1.5H_{2}O\;\left(1\right)$ was synthesized by the reaction of cobalt(II) acetate and nickel(II) acetate with HL Schiff base in an ethanolic solution (Scheme 2). HL Schiff base was synthetized as previously reported [13,14] and further characterized by X-ray diffraction analysis. Details of such characterization are reported in ESI (Table S1).

The IR spectrum of the complex has the same pattern of the well-known structure of [${\mathrm{Co}}_{4}L_{4}{\left({\mathrm{CH}}_{3}\mathrm{COO}\right)}_{2}{]}_{2}{\left[{\mathrm{BPh}}_{4}\right]}_{4}\cdot 0.5H_{2}O$ [12] and exhibits strong absorptions at 1625 and 1562 cm^−1^ assignable to the carbonyl group of the pyrazolone ring, ν(C–O), and azomethine group ν(HC=N) of “L”, respectively. The first absorption band of the complex was shifted by about 29 ${\mathrm{cm}}^{-1}$ to a lower frequency relative to the corresponding signal of the free ligand (1654 ${\mathrm{cm}}^{-1}$), indicating that the ligand coordinates through the carbonyl oxygen of the pyrazolone ring. The second absorption was shifted to the lower frequency of about 29 ${\mathrm{cm}}^{-1}$ (free ligand at 1591 ${\mathrm{cm}}^{-1}$), suggesting the involvement of the nitrogen atom of the azomethine group in the coordination. The two shoulders at 1610 and 1457 ${\mathrm{cm}}^{-1}$, can be ascribed to the asymmetric and symmetric stretching frequencies of the acetate –COO^−^ groups. The separation (143 ${\mathrm{cm}}^{-1}$) between the two absorptions indicates that the acetate group act as a bidentate bridging ligand [20]. The ν(OH) absorption at 3446 cm^−1^ indicates the presence of water molecules. The tetraphenylborate showed a finger print absorption at 2927–3044 ${\mathrm{cm}}^{-1}$. The IR spectra of complexes **1** and **2** are shown in supplementary Figures S1–S3. The UV-Vis spectrum of the title complex was obtained in acetonitrile solution. The spectrum shows absorption bands below 300 nm, a consequence of intra-ligand electronic transitions. The bands at 330 nm, 370 nm and 390 nm(sh) are assigned to the d-d transitions of the high-spin Co(II) ions in an octahedral geometry. There is a blue shift in these bands compared with the absorption spectra of some hexa-coordinate Co(II) complexes with the peak maxima wavelength (𝜆_max_) at about 500–513 nm [21]. The absorption in the visible area of the spectrum at approximately 450 nm which is not shown in the spectrum of the pure cobalt complex (**2**) (Figures S4 and S5) may be responsible for the orange color of the title complex, that can be assigned to the Ni(II) spin-allowed ^3^A_2g_(F) → ^3^T_1g_ (F) transition.

**Figure 1:**
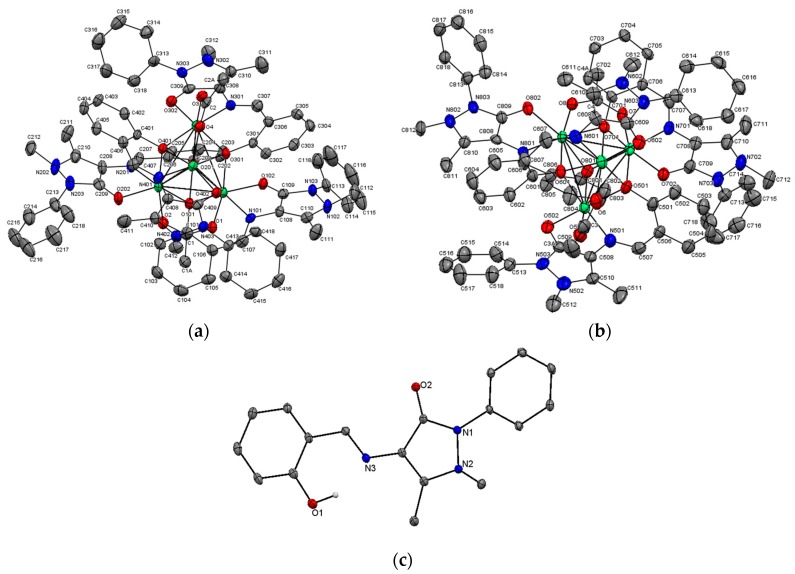
A single crystal structure of [$M_{4}L_{4}$(OAc${)}_{2}$]^2+^ cationic units (**a**) A and (**b**) B of the title complex (**1**) with 50% probability thermal ellipsoids showing the atom labeling scheme. Shared position for metal ions are shown in green color. Hydrogen atoms are omitted for clarity; (**c**) crystal structure of a ligand HL. All hydrogen atoms are omitted except phenolic hydrogen.

The crystal structure of (**1**) with atomic numbering scheme is presented as an ORTEP plot (Figure 1a,b). Relevant bond distances and angles are given in Table 2 and the view of packing diagram is shown in Figure S6 The asymmetric unit of (**1**) contains two tetranuclear cationic units, A and B having formula [$M_{4}L_{4}$(CH_3_COO${)}_{2}$]^2+^, where M = Co^2+^ or Ni^2+^, containing a cubane-like $M_{4}O_{4}$ core. In addition, four BPh_4_^−^ ions and one and half water molecule complete the crystal packing. The corners occupied by eight metal ions in the cubane-like $M_{4}O_{4}$ core are shared by cobalt(II) and nickel(II) ions in the overall stoichiometry ratio 5.33:2.67. The ratio 2:1 of Co:Ni in the mixed complex suggests that, if statistic distribution of the two ions is assumed on the four sites of each cubane, the crystal actually contains the following relative abundance of five different tetranuclear compounds: Co_4_ (19.8%); Co_3_Ni (39.5%); Co_2_Ni_2_ (29.6%); CoNi_3_ (9.9%); and Ni_4_ (1.2%). The cationic units A and B are close to be isostructural featuring only slightly differences in the corresponding bond distances and angles (Table 2). Each ligand “L” acts as a tri-chelate, coordinating the M atom by two O and one N atom. The crystal structure of the ligand HL evidenced that, while the N/phenolate oxygen bite is already preorganized, on complexation the ligand must undergo a trans, trans → cis, cis (by a rotation along its N3–C bond of the pyrazolone ring) conformational change to involve the carbonyl oxygen in metal chelation (Figure 1c). These kinds of conformational changes are known to happen in molecules containing conjugated heteroaromatic groups; a consequence of metal ions coordination [22]. The oxygen of the each phenolate group further bridges three M^2+^ ions (𝜇_3_-O) in the cubane-like core. Both cationic units contain M^2+^ ions residing in pseudo octahedral environments with MNO_5_chromophores. Each cationic unit can be regarded as a dimer of dimeric units composed by phenoxy, bridged bimetal(II) subunits. The basal-plane of each M^2+^ includes the oxygen from the phenolate group, the oxygen from the pyrazolone ring, and the azomethine nitrogen of a ligand “L”. The fourth position was occupied by the pyrazolone oxygen from another ligand. The axial sites are occupied by two oxygen atoms, one from an acetate group and the other one from the phenolate group of a third ligand “L”.

**Figure 2:**
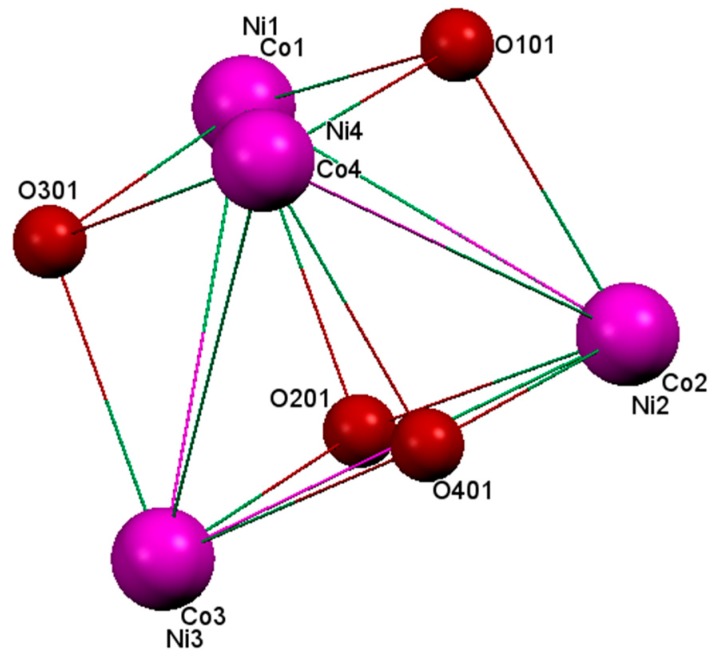
The distorted stellated octahedron composed of interleaved O-and M-based tetrahedral (unit A).

Each acetate group acts as a triatomic bidentate ligand featuring C–O bonds almost perfectly resonant, bridging two M^2+^ atoms in their familiar syn-syn manner and placed on opposite faces of the cube perpendicular to each other. The two faces bridged by an acetate group in cationic unit A exhibit shorter M·····M separations of 2.9696(7) and 3.0029(9) Å, and more acute M–O–M angles ranging from 89.7(1)° to 90.2(1)° with M–O–O–M dihedral angles of 156.4° and 157.8° compared with those not bridged by acetate group, where M·····M separations are in the range of 3.1934(8) to 3.2161(9) Å, M–O–M angles in the range of 97.1(1)° to 100.1(1)°, and M–O–O–M dihedral angles in the range of 175.7° to 179.8°. Cationic unit B has a little bit shorter M·····M separations with more acute angles (Table 2). These variations in bond distances and angles between faces of the cubane-like cores are responsible for their distortions. The $M_{4}O_{4}$ core in cationic units A and B (Figure 2) is sufficiently distorted from a regular cubic shape that it is better described as a stellated octahedron (a distortion of the stella octangula) formed by the interleaved $M_{4}{\mathrm{or}\;O}_{4}$ tetrahedra [5]. This distortion produced two types of M–O–M angles, those with value higher than 89.2(1)°, but less than 90.2(1)° placed at the top and bottom faces of the core, and those with value higher than 91.1(1)° at the rest of the cubane faces. Therefore, one can expect that the M^2+^ ions with acute angles will lead to some magnetic properties comparable to the $\left[{\mathrm{Ni}}_{4}{\left({\mathrm{OCH}}_{3}\right)}_{4}{\left(\mathrm{dbm}\right)}_{4}{\left(\mathrm{MeOH}\right)}_{4}\right]$, dbm = dibenzoylmethane [23]. The values found for bond angles, bond distances, and torsional angles in the mixed cobalt-nickel complex slightly differ from those found in the above-mentioned pure cobalt complex (Co……Co in the range of 3.24 to 3.31 Å, Co–O–Co in the range 97.26° to 102.28°, and Co–O–O–Co dihedral angles in the range 177.06° to 178.99°) [12]. This difference may be due to the presence of Ni^2+^ ions occupying part of the sites in the former complex.

The average M–OAc (OAc = CH_3_COO^−^) bond distances of complex (**1**) of 2.02 Å are longer than that reported for [${\mathrm{Co}}_{4}O_{4}$(dpah${)}_{4}$(OAc$){]}_{2}V_{4}O_{12}$·5$H_{2}$O (1.98 Å) and shorter than that reported for [N$i_{4}$(OCH_3_${)}_{4}$(OAc${)}_{2}$(TMB${)}_{4}$](BP$h_{4}{)}_{2}$·4C$H_{2}$C$l_{2}$, TMP = 2,5-dimethyl-2,5-diisocyanohexane (2.05 Å) [24,25] and very close to that reported for [${\mathrm{Co}}_{4}L_{4}{\left({\mathrm{CH}}_{3}\mathrm{COO}\right)}_{2}{]}_{2}{\left[{\mathrm{BPh}}_{4}\right]}_{4}\cdot 0.5H_{2}O$ [12]. The bond distances of M–OAc in cationic unit B are very close to that in cationic unit A. The difference in all bond distances of the acetate groups in both units ranges from 0.01 to 0.03 Å and are very close to that reported for nickel(II) ion complex [25]. The phenolate oxygen atom is ligating three metal atoms (𝜇_3_-O), the oxygen atom being 1.07–1.10 Å apart from the plane defined by the metallic triplet. The separation between metal atoms in unit A differ slightly from those ones in unit B, the longest and the shortest separation for cationic unit A are M1·····M3 3.2366(9) Å and M1·····M2 2.9696(7) Å, respectively, while for cationic unit B, they are M5·····M73.256(1)Å, and M5·····M6 2.9589(8) Å, respectively. The shortest M·····M separation in complex (**1**) is still longer than the longest one reported for [${\mathrm{Co}}_{4}O_{4}$(dpah${)}_{4}$(OAc$){]}_{2}V_{4}O_{12}$·5$H_{2}$O, dpah = 2,2-dipyridylamine (2.848 Å) [24]. Moreover, M·····M separations in both units are very close to the separation reported by Elmehdawi et al. in [${\mathrm{Co}}_{2}L_{2}$(NCS${)}_{2}$(CH_3_OH${)}_{2}$] (3.1028 Å) [26] and in [${\mathrm{Co}}_{2}L_{2}{\mathrm{Cl}}_{2}$(C_2_H_5_OH${)}_{2}$] (3.085 Å) [27], where “L” is the same Schiff base in the first and 2-hydroxyisophtaldehyde oxime in the second case. The C–O bond distances in the phenolate groups of the ligands, which act as 𝜇_3_-O bridges, range from 1.365(5) to 1.374(6) Å in cationic unit A and from 1.362(5) to 1.383(5) Å in cationic unit B. These distances are still longer than C–O distance of the phenolate group which acts as 𝜇_2_-O in [${\mathrm{Co}}_{2}L_{2}$(NCS${)}_{2}$(CH_3_OH${)}_{2}$] (1.330(2) Å) and even longer than the distance of the free Schiff base (1.345 Å).

Further analysis of the crystal structure of (**1**) revealed that the structure contains one and half water molecules in the asymmetric unit, featuring different H-bond contacts of O·····O type OW1 ·····O5 2.85 Å, O·····H3A2 (acetate methyl group) 2.880(4) Å and CH·····O type H-bonds involving the H-atoms of the benzene rings and the O-atoms of the pyrazolone rings of the Schiff bases, with the shortest C102–H·····O202 = 2.188(3) Å and the longest C418–H·····O402 = 2.675(3) Å. The four counter ions BP$h_{4}$^−^exhibit their normal geometry. We tried to prepare a nickel complex isostructural with [${\mathrm{Co}}_{4}L_{4}{\left({\mathrm{CH}}_{3}\mathrm{COO}\right)}_{2}{]}_{2}{\left[{\mathrm{BPh}}_{4}\right]}_{4}\cdot 0.5H_{2}O$ [12], from nickel acetate and the ligand “L”. This reaction ends up with the formation of [N$i_{2}$(CH_3_CO_2_)$L_{2}$(H_2_O${)}_{2}$][BP$h_{4}$]. The crystal structure of this dimer indicates that the Ni^2+^ ions are bridged by two phenoxy O-atoms from two L ligands leading to Ni_2_O_2_ rhombic ring with Ni·····Ni separation of 2.948(1) Å and Ni–O–Ni bridging angles of 91.8(2)° and 92.6(2)°. In this compound the Ni·····Ni distance is shorter and the Ni–O–Ni angles a little bit less acute than those reported for the titled complex and for its counterpart pure Co_8_-complex [28].

### Magnetic Properties

**Figure 3:**
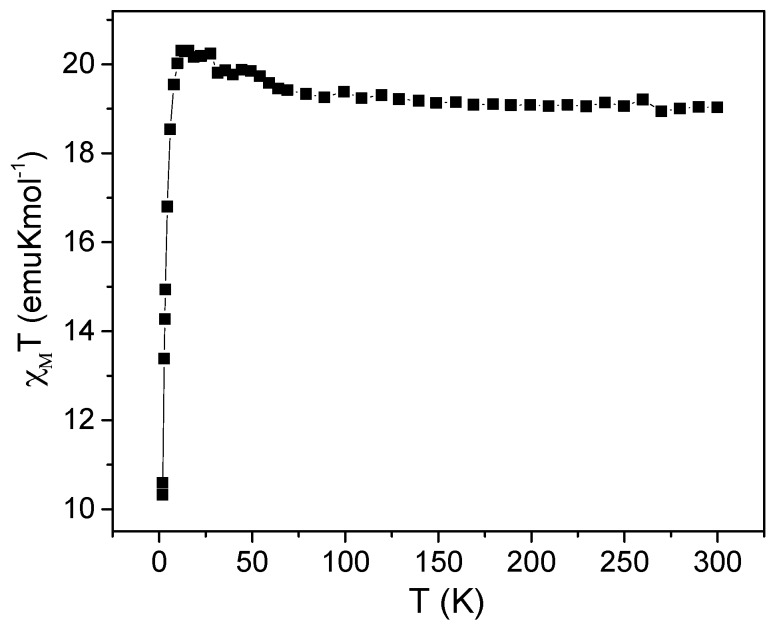
Temperature dependence of the χ_M_T product (per 5.3 cobalt and 2.7 nickel dications) of **1** observed at 1 kOe.

Magnetic direct-current (dc) susceptibility measurements in a 1 kOe and 10 kOe magnetic field were performed as a function of temperature (from 1.9 K to 300 K) on pellets of the randomly oriented polycrystalline samples obtained by grinding of single crystals of (**1** and **2**). The χ_M_T (χ_M_ is the molar paramagnetic susceptibility) νs T plot of the mixed cobalt(II)-nickel(II) complex is shown in Figure 3. At room temperature, the χ_M_T product is 19 emu·K·mol^−1^. This value is consistent with the expected value for the presence of 5.3 Co^2+^ ions (*S* = 3/2 with large orbital contribution, so that *g* ≈ 2.5) and 2.7 Ni^2+^ ions (*S* = 1, *g* ≈ 2.15) on average for each molecule. The χ_M_T product increases gradually with cooling to reach a smooth maximum of 20.5 emu·K·mol^−1^, at 5 K and decreased sharply down to 10.3 emu·K·mol^−1^ at 1.8 K. This indicates dominant ferromagnetic interactions within the two tetramer units, followed by an additional possible antiferromagnetic interaction (between the tetramers) or relatively large anisotropy effects below 5 K. It is to be noted here that, due to the mixed metal character of this compound, the measured sample has to be considered as a mixture of five possible different tetranuclear compounds: Co_4_ (19.8%); Co_3_Ni (39.5%); Co_2_Ni_2_ (29.6%); CoNi_3_ (9.9%); Ni_4_ (1.2%), making a more refined magnetic analysis essentially impossible, since interactions of different types and magnitudes can be active in different tetranuclear compounds.

**Figure 4:**
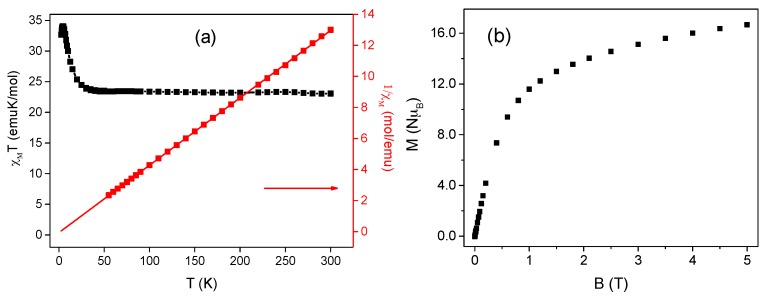
(**a**) The temperature-dependent magnetic susceptibility at 1 kOe of the pure cobalt complex. The red line represents the best fit of the Curie−Weiss law χ_M_ = C/(T − θ). The parameters are explained in the text; (**b**) Isothermal Magnetization observed at 2.5 K as a function of applied magnetic field.

For the pure cobalt complex, the room temperature χ_M_T value of 23.17 emu·K·mol^−1^ is in good agreement with the value of 23.4 emu·K·mol^−1^ expected for eight isolated Co^2+^ ions with largely unquenched orbital contribution, each with (*S* = 3/2, *g* ≈ 2.5, 3.14 emu·K·mol^−1^ as shown in Figure 4a. On cooling, χ_M_T product remains essentially constant till 35 K, followed by a monotonic increase till 5 K, showcasing a maximum value of 35 emu·K·mol^−1^. This behavior can be attributed to the ferromagnetic interactions in cubane as the Co–Co distance is small (≈ 3 Å) and the oxygen bridges are efficient in transmitting the interaction. We reiterate here that at low temperature each Co(II) ion can be treated as an effective doublet with large effective g value, due to the combined action of low symmetry distortion and spin-orbit coupling on the ^4^T_1g_ ground state in octahedral symmetry. It follows that the observed maximum χ_M_T value is consistent with four doublet interacting ferromagnetically, with an effective g value of 4.8, within the expected range [29,30]. Interestingly, a decrease in χ_M_T approaching zero is observed below 5 K down to the lowest available temperature. This decrease in χ_M_T at low temperature can be interpreted as a consequence of global/inter-tetramer antiferromagnetic interactions and/or a result of zero-field splitting usually associated with anisotropic high-spin Co^2+^ ions [31]. Due to the large number of parameters involved, we did not attempt a fit of the magnetic data, which would have provided meaningless, and highly correlated, parameter values. The temperature dependence of χ_M_^−1^ is also shown in Figure 4a. 1/χ_M_ obeys the Curie−Weiss law with C = 23.04 ± 0.02 emu·K·mol^−1^ and θ = 1.42 ± 0.12 K (solid red line), revealing ferromagnetic interactions between the Co^2+^ ions and the presence of large orbital contribution as predicted [32].

The isothermal magnetization as a function of external dc magnetic field of the pure cobalt complex was also studied. At 5 T and 2.5 K, no saturation of magnetization is observed, reaching a maximum value of 16.63 Nμ_B_ as shown in Figure 4b. While this value is apparently inconsistent with that associated with eight parallel oriented high spin Co^2+^ spins (*S* = 3/2, *g* = 2.5) one should again consider the peculiar electronic structure of octahedrally distorted cobalt(II) ions. For the two tetranuclear compounds made up of ferromagnetically coupled centers, and assuming the value of *g* = 4.8 derived by the χ_M_T maximum, a magnetization saturation value of 19.2 Nµ_B_ is expected. The slightly lower experimentally observed molar magnetization concurs with the aforementioned antiferromagnetic coupling within the two cobalt tetramers and with the large anisotropy associated with cobalt [33]. The magnetic dynamic measurements were also carried out as a function of frequency, dc magnetic field, and temperature on the pure cobalt complex. No out-of-phase (*χ*_M_″) alternating current (ac) susceptibility component was observed in zero dc field. Whereas in non-zero field, although a non-zero χ_M_″ signal was observed, it lacked a clear maximum as a function of frequency, thus indicating that the complex does not show a typical SMM behavior as shown in supplementary Figure S7.

## 4. Conclusions

We conclude that the mixed cobalt(II)/nickel(II) complexes, which have two A and B cationic units are almost isostructural with its counterpart pure cobalt complex. Each alternate corner of a cubane in complex (**1**) is shared by Co^2+^ and Ni^2+^ ions in the overall stoichiometry ratio, for the two cubane moieties, of 5.33:2.67. The distortion of the cubane like units lead to some magnetic interactions. The magnetic characterization of the new complex indicates ferromagnetic interactions within the two tetramer units, as expected by geometrical considerations, followed by an additional weak antiferromagnetic interaction between the tetramers. Ferromagnetic interaction is observed within the cubane for both complexes. The pure Co complex does not show signatures of SMM behavior.

## Figures and Tables

**Table 1:** Crystal data and structure refinement details for (**1**).

Empirical Formula	C_248_H_223_B_4_Co_5.33_Ni_2.67_N_24_O_25.50_
Formula Weight	4461.58
Temperature	120 K
Wavelength	0.71073 Å
Crystal System	Triclinic
Space Group (Number)	P-1 (2)
Unit Cell Parameters	*a* = 19.2237(9) Å, *α* = 97.630(4)°
	*b* = 21.966(1) Å, *β* = 107.410(4)°
	*c* = 28.988(1) Å, *γ* = 109.075(4)°
Cell Volume	10672.1(9) Å^3^
*Z*	2
*ρ*c	1.388 g·cm^−3^
*μ*	0.71 mm^−1^
*F* (000)	4643
Crystal Size	0.05 × 0.04 × 0.03 mm
*θ* max for Data Collection	25°
Reflections Collected	113258
Independent Reflections	36372 [(int) = 0.099]
Completeness	99.6%
Data/Restraints/Parameters	36,372/0/2867
Goodness-of-Fit on *F*^2^	0.727
*R* Indices	[*I* > 2*σ*(*I*)] *R*1 = 0.0497, *wR*2 = 0.0757
*R* Indices (All Data)	*R*1 = 0.1557, *wR*2 = 0.0881

**Table 2:** Selected bond lengths (Å) and angles (°) of (**1**) where site occupancy factors (s.o.f.) for M are 0.67 for (Co) and 0.33 for (Ni) Bond lengths.

Bond	Length	Bond	Length	Bond	Length
M1–O101	2.084(2)	M1–O102	2.129(2)	M1–O201	2.120(3)
M1–N101	2.059(4)	M1–O301	2.118(3)	M1–O1	2.006(3)
M2–O101	2.125(4)	M2–O201	2.072(2)	M2–N201	2.061(4)
M2–O202	2.106(2)	M2–O401	2.159(4)	M2–O2	2.005(3)
M3–O201	2.123(3)	M3–N301	2.077(3)	M3–O301	2.077(3)
M3–O401	2.131(2)	M3–O302	2.100(3)	M3–O3	2.035(3)
M4–O101	2.120(3)	M4–O301	2.162(2)	M4–N401	2.057(3)
M4–O401	2.069(3)	M4–O402	2.107(3)	M4–O4	2.011(3)
M5–O501	2.070(4)	M5–N501	2.0525(3)	M5–O502	2.069(4)
M5–O601	2.138(2)	M5–O801	2.132(3)	M5–O5	2.033(3)
M6–O501	2.129(2)	M6–O601	2.074(3)	M6–O602	2.084(4)
M6–N601	2.074(3)	M6–O701	2.124(3)	M6–O6	2.030(4)
M7–O501	2.188(3)	M7–O701	2.084(2)	M7–N701	2.071(46)
M7–O702	2.119(2)	M7–O801	2.123(3)	M7–O7	2.024(3)
M8–O601	2.112(3)	M8–O701	2.132(2)	M8–O801	2.092(2)
M8–N801	2.069(4)	M8–O802	2.144(2)	M8–O8	1.990(3)
Bond Angles					
M1–O101–M2	89.7(1)	M1–O101–M4	98.9(1)	M2–O101–M4	99.3(1)
Bond Angles					
M1–O101–M2	89.7(1)	M1–O101–M4	98.9(1)	M2–O101–M4	99.3(1)
M1–O201–M3	99.4(1)	M1–O201–M2	90.2(1)	M2–O201–M3	100.1(1)
M1–O301–M3	100.8(1)	M1–O301–M4	96.5(1)	M3–O301–M4	90.0(1)
M2–O401–M3	97.1(1)	M2–O401–M4	99.9(1)	M3–O401–M4	91.3(1)
M5–O501–M6	89.6(1)	M5–O501–M7	99.7(1)	M6–O501–M7	97.8(1)
M5–O601–M6	89.2(1)	M5–O601–M8	97.4(1)	M6–O601–M8	101.2(1)
M6–O701–M7	101.3(1)	M6–O701–M8	98.9(1)	M7–O701–M8	89.6(1)
M5–O801–M7	99.9(1)	M5–O801–M8	98.1(1)	M7–O801–M8	89.6(1)

## Supplementary Materials

The following are available online at www.mdpi.com/1996-1944/10/2/178/s1. Figure S1: The IR spectrum of the ligand HL, Figure S2: The IR spectrum of mixed cobalt/nickel, complex **1**, Figure S3: The IR spectrum of [Co_4_L_4_(CH_3_COO)_2_]_2_[BPh_4_]_4_, complex **2**, Figure S4: View of a Pack diagram of [${\mathrm{Co}}_{5.33}{\mathrm{Ni}}_{2.67}L_{8}{\left({\mathrm{CH}}_{3}\mathrm{COO}\right)}_{4}]{\left[{\mathrm{BPh}}_{4}\right]}_{4}\cdot 1.5H_{2}O.$ Hydrogen atoms are omitted for clarity, Figure S5: UV-Vis spectrum of mixed Co(II)/Ni(II) complex **1**, Figure S6: UV-Vis spectrum of pure Co(II) complex **2**, Figure S7: (a) Dc magnetic field and frequency dependence of the imaginary component of the ac susceptibility observed at 2 K. (b,c) In and out-of-phase ac susceptibility components observed at as a function of temperature in fixed 1 kOe field, Table S1: Crystallographic data and refinement parameters for HL.
