# Supplementary material for: Synthesis, Crystal Structure, and Magnetic Properties of a New Mixed Metal (Co(II), Ni(II)) Cubane
Source: Materials (Basel). 2017 Feb 14;10(2):178. doi: 10.3390/ma10020178 (PMC5459200; doi:10.3390/ma10020178)
Supplement: Supplementary file 1 [file materials-10-00178-s001.pdf]

Supplementary Materials: Synthesis, Crystal Structure, and Magnetic Properties of a New Mixed Metal (Co(II), Ni(II)) Cubane

Ramadan Mohamed Elmehdawi, Mohamed Nasir EL-Kaheli, Ramadan Gamodi Abuhmaiera, Fathia Ali Treish, Mufida El Mabruk Ben Younes, Carla Bazzicalupi, Annalisa Guerri, Andrea Caneschi and Asma Amjad

Details of the Crystal Structure Determination and Refinement of HL

Suitable yellow single crystals of the complex were obtained by slow evaporation of a diluted hot ethanolic solution. A prism shaped crystal of appropriate size was selected for data collection. Reflection data for the X-ray analysis of (**1**) were collected on an Oxford Diffraction Xcalibur, Sapphire3 diffractometer equipped with a Charged Coupled-Device (CCD) area detector and MoK_{α} radiation (0.71073 Å) at 100 K. Data collection, reduction, and absorption correction (SCALE3 ABSPACK routine) were carried out through the suite CrysAlysPro [1]. The structure of the complex was solved by direct methods of the SHELXD program [2] and then refined by full-matrix least squares against F^2 using all data (SHELX-2014) [3]. All non-hydrogen atoms were anisotropically refined. Phenolic hydrogen was found in the ΔF map and freely refined with isotropic displacement parameter. All the other hydrogen atoms were introduced in calculated positions with isotropic displacement parameters refined accordingly to the linked atoms. Molecular plots were produced with Ortep-3 for windows program [4]. CIF file is available from the Cambridge Crystallographic Data Center (1520222).

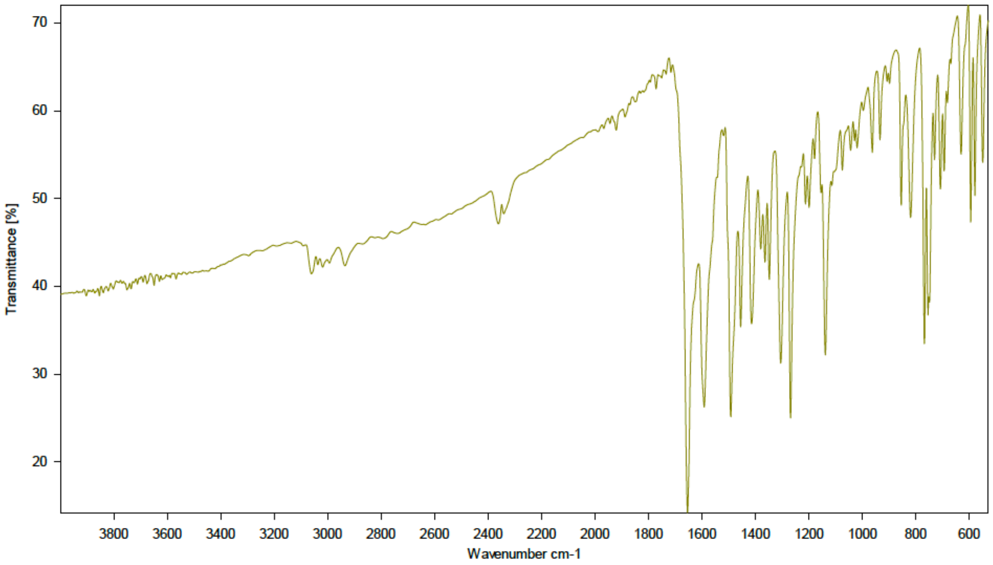

Figure S1. The IR spectrum of the ligand HL.

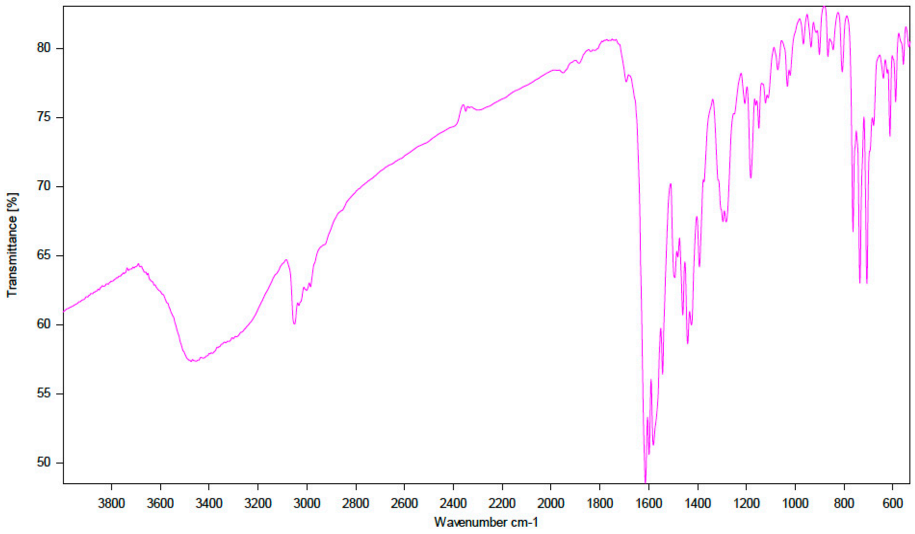

Figure S2. The IR spectrum of mixed cobalt/nickel, complex **1**.

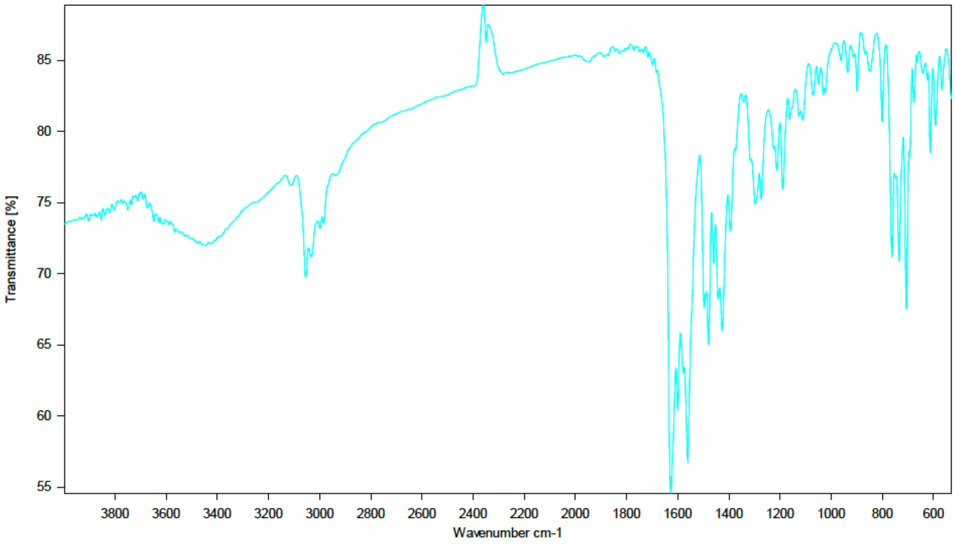

Figure S3. The IR spectrum of $[Co_4L_4(CH_3COO)_2]_2[BPh_4]_4$, complex **2**.

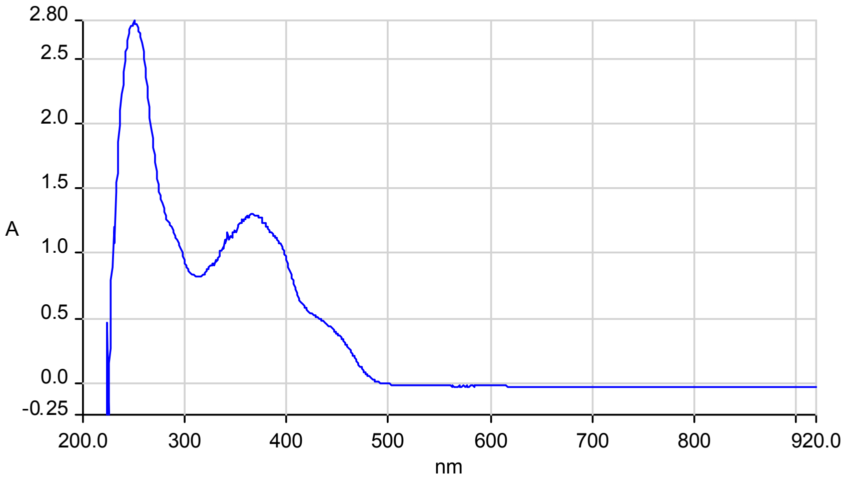

Figure S4. UV-Vis spectrum of mixed Co(II)/Ni(II) complex **1**.

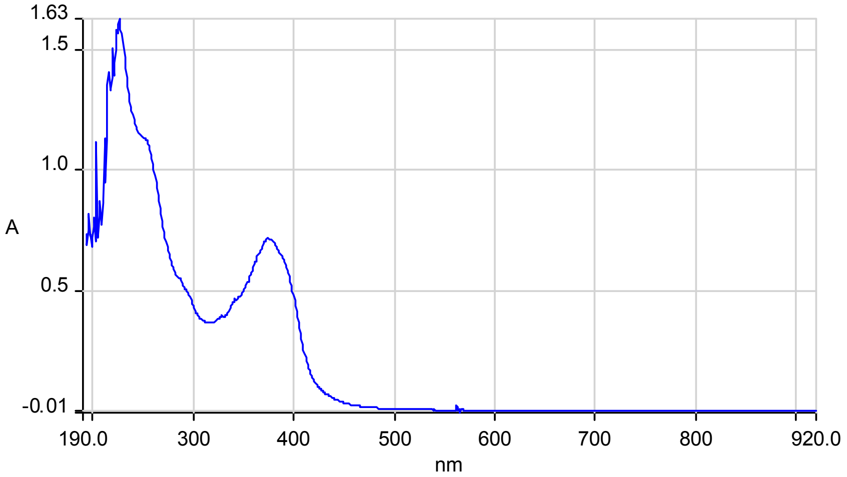

Figure S5. UV-Vis spectrum of pure Co(II) complex **2**.

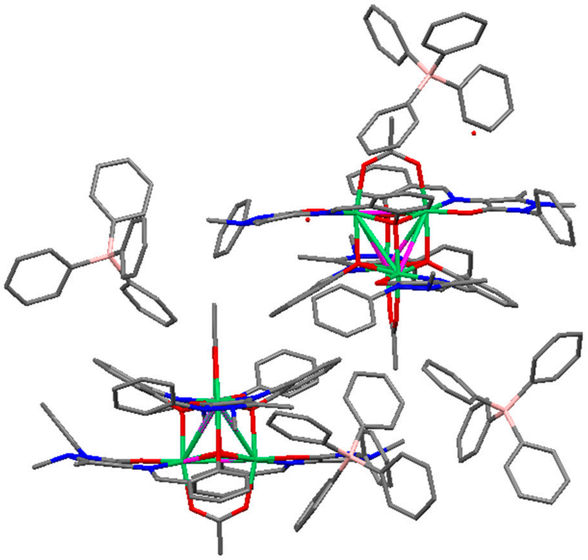

Figure S6. View of a Pack diagram of $[Co_{5.33}Ni_{2.67}L_8(CH_3COO)_4][BPh_4]_4 \cdot 1.5H_2O$. Hydrogen atoms are omitted for clarity.

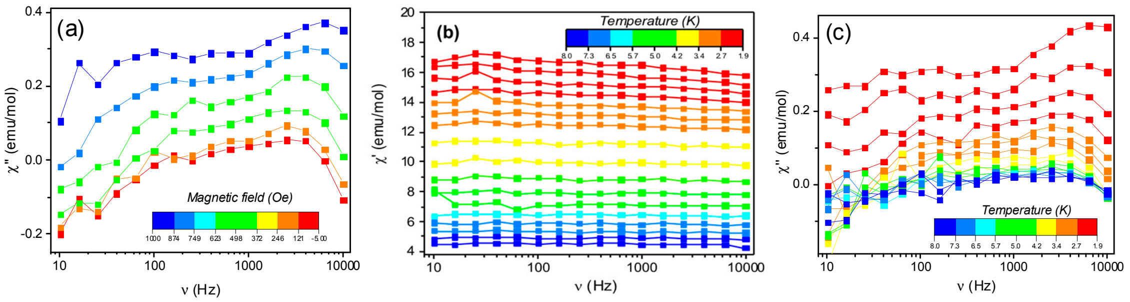

Figure S7. (**a**) Dc magnetic field and frequency dependence of the imaginary component of the ac susceptibility observed at 2 K; (**b**,**c**) In and out-of-phase ac susceptibility components observed as a function of temperature in fixed 1 kOe field.

Table S1. Crystallographic data and refinement parameters for HL.

Parameters	HL
Empirical Formula	$C_{18}H_{17}N_3O_2$
Formula Weight	307.351
Temperature (K)	100
Wavelength (Å)	0.71073
Crystal System, Space Group	Monoclinic, $P\ 2_1/n$
Unit Cell Dimensions (Å, °)	a = 7.4181(4), b = 7.4503(3), c = 27.236(1) β = 95.544(4)
Volume (Å^3)	1498.2(1)
Z, ρc(mg/cm^3)	4, 1.3615
μ (mm^{-1})	0.091
F(000)	648
Crystal Size (mm)	0.2 × 0.2 × 0.1
θ Range (°)	4.262 to 25.350
Reflections Collected/Unique	13794/2706
Data/Restraints/Parameters	2706/0/214
Goodness-of-fit on F2	1.067
Final R Indices [I > 2σ(I)]	R1 = 0.0478, wR2 = 0.0964
R Indices (All Data)	R1 = 0.0681, wR2 = 0.1049

References

1. CrysAlis PRO, Version 1.171.34.44 (release 25–10–2010 *CrysAlis171*. NET). Oxford Diffraction Ltd.: Oxfordshire, UK, 2010.
2. Schneider. T.R.; Sheldrick. G.M. Substructure solution with SHELXD. *Acta Crystallogr. D Biol. Crystallogr.* **2002**, *58*, 1772–1779.
3. Sheldrick, G.M. Crystal structure refinement with SHELXL. *Acta Crystallogr. C Struct. Chem.* **2015**, *71*, 3–8.
4. Farrugia. J.L. ORTEP-3 for Windows—A version of ORTEP-III with a Graphical User Interface (GUI). *J. Appl. Crystallogr.* **1997**, *30*, 565.

© 2016 by the authors. Submitted for possible open access publication under the terms and conditions of the Creative Commons Attribution (CC-BY) license (http://creativecommons.org/licenses/by/4.0/).
